# Are Vitamin E Supplementation Beneficial for Female Gynaecology Health and Diseases?

**DOI:** 10.3390/molecules27061896

**Published:** 2022-03-15

**Authors:** Nur Amira Md Amin, Siti Hamimah Sheikh Abdul Kadir, Akmal Hisyam Arshad, Norhaslinda Abdul Aziz, Nurul Alimah Abdul Nasir, Normala Ab Latip

**Affiliations:** 1 Institute for Pathology, Laboratory and Forensic Medicine (I-PPerForM), Universiti Teknologi MARA, Cawangan Selangor, Sungai Buloh 47000, Selangor, Malaysia; 2020721353@student.uitm.edu.my; 2 Institute of Medical Molecular Biotechnology (IMMB), Faculty of Medicine, Universiti Teknologi MARA, Cawangan Selangor, Sungai Buloh 47000, Selangor, Malaysia; 3 Department of Biochemistry, Faculty of Medicine, Universiti Teknologi MARA, Cawangan Selangor, Sungai Buloh 47000, Selangor, Malaysia; 4 Department of Obstetrics and Gynaecology, Faculty of Medicine, Universiti Teknologi MARA, Sungai Buloh 47000, Selangor, Malaysia; akmalhisyam@uitm.edu.my; 5 Department of Obstetrics and Gynaecology, Faculty of Medicine, Universiti Kebangsaan Malaysia Medical Centre, Cheras 56000, Kuala Lumpur, Malaysia; norhaslinda.abdaziz@ppukm.ukm.edu.my; 6 Department of Pharmacology, Faculty of Medicine, Universiti Teknologi MARA, Cawangan Selangor, Sungai Buloh 47000, Selangor, Malaysia; nurulalimah@uitm.edu.my; 7 Atta-ur-Rahman Institute for Natural Products Discovery (AuRIns), Faculty of Pharmacy, Universiti Teknologi MARA, Cawangan Selangor, Puncak Alam 42300, Selangor, Malaysia; drnormala6351@puncakalam.uitm.edu.my

**Keywords:** vitamin E, tocotrienol, tocopherol, gynaecology health, gynaecology disease

## Abstract

Vitamin E is known as an essential vitamin, and many studies had demonstrated the importance of vitamin E throughout the reproductive process, such as miscarriage, premature birth, preeclampsia, and intrauterine growth restriction, which could be caused by a lack of vitamin E during pregnancy. Its potent antioxidant properties can counteract the oxidative stress induced by oxygen free radicals and imbalance of oxidative-antioxidant levels, hence it may play a role in maintaining the normal function of the female reproductive system. Despite the fact that vitamin E is acknowledged as the substance needed for reproduction, its beneficial effects on female fertility, gynaecological health, and diseases are still poorly understood and lacking. Therefore, the goal of this paper is to provide a summary of the known roles of vitamin E supplementation in women for gynaecological health and reproductive-related diseases, as well as its future perspective.

## 1. Introduction

Evans and Bishop were the first to discover vitamin E, reporting that it was an essential vitamin for rat reproduction [1]. Subsequently, Olcott et al. discovered its function as a powerful lipid antioxidant agent [2]. Up till now, vitamin E has been the subject of increasingly in-depth research to understand its role and effects. Tocopherols (TCPs) and tocotrienols (TCTs) are the two naturally occurring forms of vitamin E, and each group has four distinct structural isomers, namely alpha (α), beta (β), gamma (γ), and delta (δ). TCPs are saturated forms of vitamin E, whereas TCTs are unsaturated with an isoprenoid side chain. Vitamin E obtained from natural sources is only present in the R-form (dextral); TCPs possess R at positions 2, 4, and 8 (RRR-form), whereas TCTs possess R only at position 2 (R-form) (Table 1). The other S-form (sinistral) can be found in synthetic vitamin E [3].

There is no official guidelines for vitamin E intake, and there is no evidence of toxicity, when obtained only from dietary sources [4]. The recommended dietary allowance (RDAs) for vitamin E is 15 mg/day (Table 2), but many supplements contain significantly greater daily doses, ranging from 100 to 1000 mg [5]. When taken in excess, vitamin E toxicity may cause major bleeding events. In most individuals, symptoms do not manifest until daily doses exceed 1000 mg [6]. It has also long been known that a high concentration of α-TCP has the capacity to behave as a pro-oxidant and accelerate the peroxidation of lipids in in vitro studies [7,8]. Similarly, recent in vitro studies on γ-TCT have shown that it may act as a pro-oxidant and induce toxicity at high concentrations [9,10].

Despite having similar structures and antioxidant activities, each form differs greatly in their bioavailability and metabolism [11]. In general, the absorption of vitamin E follows the same pathway as dietary fats; both TCPs and TCTs are absorbed, in the form of chylomicrons, from the intestinal lumen and transported to the peripheral tissues through lymphatic system [12]. However, it has been shown that bioavailability of TCTs is relatively lower than that of TCPs. Due to the fact that TCPs have a higher binding affinity for the major vitamin E transport protein, alpha-tocopherol transfer protein (α-TTP), hence TCTs must compete with TCPs for binding to α-TTP. As a consequence, the concentration of TCTs in the plasma is reduced [13]. Between TCPs, α-TTP exhibits 100% affinity for α-TCP, 38% for β-TCP, 9% for γ-TCP, and 2% for δ-TCP [14]. When comparing the bioavailability between TCTs, α-TCT has the highest oral bioavailability and it can be absorbed more readily when there are fewer or absence of TCPs in the dietary matrix [15]. Aside from that, vitamin E dispersion in the intestinal lumen, along with other dietary fats, such as fatty acids and plant sterols, may significantly affect the bioavailability of vitamin E [16].

Vitamin E metabolism, which takes place mostly in the small intestine and liver, involves a series of enzymatic processes that are similar for all four TCPs and TCTs [17]. The liver, on the other hand, is crucial for vitamin E metabolism; since only the liver expresses α-TTP, thus favouring α-TCP over other forms of vitamin E [18]. This is an important mechanism that prevents excessive α-TCP breakdown and excretion, while also regulating the level of non-α-TCP forms in circulation [19]. The liver predominantly releases α-TCP by incorporating it into very low density lipoprotein (VLDL), while the non-α-TCP forms are immediately metabolized, followed by their excretion [20,21,22]. Even though the rate of catabolism is faster for TCTs than in TCPs and varies among the TCPs, their metabolic pathway is the same [23]. In humans, cytochrome P 450 (CYP), primarily CYP4F2/CYP3A4, regulates the first and rate-limiting stage of vitamin E metabolism, which, hence, controls the formation of the vitamin’s breakdown products. Carboxyethyl-hydroxychromanols (CEHC), also known as 3′-COOH or short-chain metabolites (SCM), are the final products of vitamin E metabolism [24] and serve as the useful biomarkers of its intake [25]. Vitamin E is excreted in two main pathways; the main route of excretion is bile, followed by urine. Due to its poor intestinal absorption, vitamin E is mostly excreted through faeces [24,26]. Vitamin E can be found naturally in foods that are rich in fats, such as nuts, seeds, and vegetable oils [27]. All of the forms of vitamin E can be found in edible vegetable oils in varied proportions, depending on their source (Table 3) [28]. Vegetable oils, such as soybean, sunflower, and almond oils, have high concentration of TCPs [29], while palm, rice bran, and coconut oils are rich in TCTs [30,31,32]. In rice, palm, and annatto oils, the ratio of TCTs to TCPs are 50:50, 75:25, and 99.9:0.1, respectively [33]. Palm oil, extracted from the fruits of Elaeis guineensis, has a relatively high level of TCTs, and only palm oil contains complete homologs of TCTs. After esterification and subsequent distillation, crystallisation, and chromatography of crude palm oil, it will yield around 27.30% of α-TCT; 3.34% of β-TCT, 35.51% of γ-TCT, and 10.45% of δ-TCT [34].

Before, the 1980’s studies on vitamin E were mostly done on TCPs and its antioxidant properties; however, during the late 1980’s, the focus has changed to TCTs, when their cholesterol-lowering [36] and anticancer properties were reported [37,38,39]. Later, a great number of experimental research and clinical trials demonstrated the effectiveness of TCTs towards numerous diseases, such as skin aging [40,41], diabetes [42,43], neurodegenerative [41,44], and cardiovascular diseases [45,46]. Since then, new insight of TCTs, as a potent anti-inflammatory and antioxidant, were discovered [47], and the potential benefit of TCTs in female gynaecology health and reproductive-related diseases were reported.

Therefore, this review article aims to summarise the various potentials of vitamin E and its isomers: (i) its significance in female reproductive and gynaecological health; (ii) its effects on female reproductive hormones; (iii) its role in assisted reproductive technologies (ART); (iv) its effects on gynaecological infections and related diseases; and (v) its effects on gynaecological cancer. The literature search was conducted between March and February 2022, in PubMed and Google Scholar, for articles related to vitamin E, gynaecology health, and diseases, using specific keywords, such as tocopherol, tocotrienol, vitamin E, pregnancy, reproductive hormones, and diseases. In terms of diseases, the focus was mainly on the most often discussed diseases, such as pregnancy-related diseases, infections, and gynaecological cancer. This review includes clinical trials, controlled trials, systematic reviews, and meta-analyses.

## 2. Vitamin E in Female Fertility, Pregnancy, and Reproductive Health

Female fertility refers to a woman’s ability to conceive, carry a pregnancy to term, and give birth to a child. Infertility is when a woman is not able to conceive after one year of trying to get pregnant, or six months for woman of 35 years old or older. The physiological process of reproduction is very complex, and problems at any of these stages can lead to infertility. The mechanism of how vitamin E plays a role in any of these physiological processes in still unclear, but several studies have revealed that vitamin E could have a promising potential effect that can improve the rate of female fertility and reproductive health [48,49,50,51].

Vitamin E’s antioxidant properties may protect both the mother and baby throughout pregnancy by acting as a chain-breaking antioxidant and the body’s primary lipid peroxyl radical scavenger, hence reducing the likelihood of complications during pregnancy [52]. Supplementation of vitamin E (400 IU/day) and vitamin C (1000 mg/day) women at increased risk of pregnancy complications may help to reduce the incidence of oxidative stress-related pregnancy complications, such as pre-eclampsia [53,54]. The oxidative stability of vitamin E levels in maternal blood was demonstrated to be increased throughout normal pregnancies [55]. Research has also shown that the metabolism of vitamin E changes during pregnancies. According to the study, vitamin E levels are lower in abnormal pregnancy, compared to normal pregnancy, with a mean vitamin E concentration increased from 12.9 μg/mL in early pregnancy to 22.5 μg/mL at term in normal pregnancies [56]. Another study also indicated that vitamin E is critical for female reproductive health when higher vitamin E levels are stated to be required to neutralize the free radicals present from smoking cigarette [57]. Additionally, there has been a substantial drop in vitamin E levels, as well as other antioxidant vitamins, in the blood plasma of women who have recurrent pregnancy loss (RPL) [58]. On top of that, deficiency in vitamin E may lead to infertility and severe degenerative disorders, such as ataxia and Duchenne muscular dystrophy muscle degeneration [33]. A recent study also found that vitamin E levels were markedly higher in patients with gestational diabetes mellitus (GDM), a common complication of pregnancy, than in healthy pregnant women, therefore indicating that vitamin E and oxidative stress levels play a significant role in patients with GDM [59]. According to these findings, vitamin E is necessary for a good and healthy pregnancy.

Vitamin E treatment was also tested on women with unexplained infertility who were undergoing controlled ovarian stimulation and intrauterine insemination (IUI) [48]. In this trial, Group A (*n* = 53) had controlled ovarian stimulation, with clomiphene citrate and 400 IU/day of vitamin E (α-TCP) supplementation, whereas control Group B (*n* = 50) had ovulation induction alone. The trial’s findings revealed a significant difference in the thickness of endometrial on the day of human chorionic gonadotropin (hCG) administration between the two groups; however, no significant correlation observed between supplementation of vitamin E and the rates of implantation and pregnancy. Based on their findings, they suggested that vitamin E, through its antioxidant action, may enhance the endometrial environment and thickness in women with unexplained or idiopathic infertility, as well as modify the anti-estrogenic effect of clomiphene citrate [48]. Another study also supported this finding and suggested that the supplementation of vitamin E showed improvements in the thickness of endometrial, levels of plasma MDA, and LDLR, IL-1, and TNF-α gene expression in women with implantation failure [60].

More detailed experiments, using animal models in vivo, was done by Khanna et al.; in this study, tocopherol transport protein (TTP) deficient mice were fed with α-TCT and bred to investigate tissue delivery of oral α-TCT [49]. The findings of their study revealed that α-TCT was effectively delivered to numerous important organs and successfully restored the fertility of mice. As a result, it was hypothesized that α-TCT may sustain reproductive function in the absence of α–TCP [49]. Another study, using palm oil tocotrienol-rich fraction (TRF), found that co-administration with 5 mg/kg body weight (bw) of nicotine and 60 mg/kg of TRF can counteract nicotine-induced retarded embryogenesis and pregnancy loss in rats by raising the pregnancy rate to 83.3% [51] 11 March 2022 02:46:00. In addition, another study indicates that daily dosage of 5.0 mg/kg/day nicotine for 7 days stops in vitro preimplantation embryo development; however, concomitant treatment of 60 mg/kg/day of γ-TCT, concurrently with nicotine, consistently maintains in vitro embryo development [61]. At the optimum doses, supplementation of γ-TCT could efficiently reverse the detrimental effect of nicotine on in vitro embryo development in mice by suppressing the level of oxidative stress [61]. Overall, these findings evidently demonstrate that vitamin E has significant potential in regulating reproductive processes. Yet, the knowledge involving the significance of vitamin E in aiding reproductive success molecularly is, indeed, lacking, and more research is needed to uncover the underlying process.

## 3. Vitamin E and Reproductive Hormones

Female reproductive hormones, such as estrogen and progesterone, are usually made in the ovaries; they are responsible for developing and maintaining female sex characteristics and play essential role in the menstrual cycle, fertility, and pregnancy. Estrogen and progesterone are primarily important for regulating the endometrial tissue growth by stimulating and inhibiting cell proliferation. Even though it is the natural role of estrogen, it can also raise a woman’s risk of getting endometrial and breast cancer [62]. Concerns have been raised about the impact of vitamin E on the environment of female hormones, since it possesses oestrogenic, androgenic, and progesterone-like properties that can act in collaboration with ovarian hormones and testosterone [63]. However, no report on the direct effects of vitamin E or its isomers on female reproductive hormones is available. Available studies focus only on the correlation between exposure to multiple environmental contaminants that affect the production of oestrogen, androgen, and other hormonal pathways, as well as increased reactive oxygen species (ROS)-induced oxidative stress (OS) [64,65,66,67]. Increased OS levels in the peritoneal cavity may be associated with a number of pregnancy-related complications, including early spontaneous abortion, embryonic death, intrauterine growth restriction, foetal death, preterm delivery, and low birth weight [68,69]. Certainly, additional research is required to unravel the molecular pathways linking ROS generation, antioxidant agents, and reproductive hormones, in order to gain a better understanding of the possible involvement vitamin E may have in regulation of female reproductive hormones.

## 4. Vitamin E Supplementation on Pregnancy Outcome in Assisted Reproductive Technologies

Assisted reproductive technology (ART) is a frequent treatment option for couples having infertility issues, whether caused by male or female factors or idiopathic factors. However, it is important to note that the application of ART technology comes with its own set of challenges. This is because the in vitro setting is not as optimal as the in vivo environment, where the endogenous antioxidant system regulates the ROS build-up that results in OS [70]. While physiological levels of ROS are necessary for optimal reproductive function in vivo, the manipulation of gametes and embryos in vitro exposes these cells to exogenous or endogenous environmental stimuli that cause excessive ROS generation [71]. IUI and in vitro fertilisation (IVF) are the most often performed ART procedures in fertility treatment to increase a couple’s chance of becoming pregnant [71,72]. Comparing the two procedures, it was reported that IUI should be the primary treatment choice for the majority of couples, except in situations with blocked fallopian tubes and severe oligozoospermia [73]. Recently, there has been an increase in the data supporting the relevance of micronutrients in improving fertility rates in couples [74,75,76,77,78], therefore suggesting that vitamin E supplementation might have positive potential outcomes on couples that are undergoing ART therapy.

High-quality oocytes and maturation are critical in IVF and intracytoplasmic sperm injection (ICSI) to achieve high fertilisation and implantation rates [79,80]. However, ovarian stimulation, which is an important first step in IVF/ICSI, causes a disturbance in the oxidant-antioxidant ratio, which leads to OS [81]. An increase in OS may result in impaired oocyte maturation and a greater likelihood of IVF/ICSI cycle failure [80,82]. According to a study conducted by Bahadori et al., there is a favourable association between the vitamin E levels in follicular fluid (FF) and serum with oocyte maturation and embryo quality in women undergoing IVF [83]. According to their findings, vitamin E levels of 0.35/1 mg/dL and 1.5/2 mg/dL were shown to be the optimal concentration in FF that allowed for the highest percentage of metaphase II oocytes, while 10/15 mg/dL was associated with a high percent of higher quality of embryos achieved [83]. Additionally, a recent study on the effects of pentoxifylline (PTX) and vitamin E (TCP) on pregnancy rate in infertile women treated with ICSI found that supplementation with PTX (400 mg twice/day) and vitamin E (400 IU twice/day), for a period of 3 months prior to the embryo transfer cycle, markedly increased the thickness of endometrial and rate of successful pregnancy [84].

Overall, supplementation of vitamin E in women has been shown to have good impact in ART therapy outcomes. Nonetheless, more clinical trials on the role of vitamin E on the success of ART therapy are needed to back up these findings, due to a lack of clinically significant outcomes, such as pregnancy and live birth rates.

## 5. Vitamin E in Female Gynaecology Diseases

Gynaecological diseases are those that affect a woman’s reproductive system. These diseases include benign and malignant tumours, pregnancy-related diseases, infections, and endocrine diseases. Common gynaecological issues are endometriosis, dysmenorrhea, and genital tract infections. Vitamin E that has potent antioxidant and anti-inflammation properties that have been shown to be helpful in the treatment of several gynaecological diseases.

In order to evaluate the efficacy of antioxidants properties of vitamin E and C in the treatment of pelvic pain related to endometriosis, Kavtaradze et al. performed a clinical trial in 59 patients (age ranging from 19 to 41 years old) who experienced pelvic discomfort and had a history of endometriosis and/or infertility. The patients were randomly assigned to receive either vitamin E (1200 IU) and C (1000 mg) (group A) or a placebo (group B) daily for two months, and the pain scores were administered on a monthly basis during the study period. The patients were divided into two groups, and vitamin E (1200 IU) and C (1000 mg) (group A) or placebo (group B) were prescribed randomly (daily for a period of two months), and the pain scales were administered on a monthly basis, while on medication. According to the results of this clinical trial, women with endometriosis who were given both vitamin E and C experienced less pelvic discomfort, showing that antioxidant vitamins are effective in decreasing chronic pelvic pain [85]. Similarly, in a separate randomized trial performed by Mier-Cabrera et al., women with endometriosis who received vitamin E (α-TCP from sunflower seeds and peanuts) and C supplements had higher levels of enzymatic antioxidants, superoxide dismutase, and glutathione peroxidase, as well as nonenzymatic antioxidants, and vitamins E and C in their plasma [86].

In another research, 120 women with primary dysmenorrhea were assigned randomly to one of two groups, and 94 women completed the study. During two consecutive cycles, the treatment group (*n* = 42) received 400 IU/day of vitamin E, commencing 2 days before menstruation and lasting for a total of 5 days, whereas the control group (*n* = 52) was given a placebo. According to their findings, women with primary dysmenorrhea who took vitamin E experienced a greater reduction in pelvic pain [87]. An earlier study also found that vitamin E can significantly lessen the severity and length of discomfort associated with primary dysmenorrhea, as well as menstrual blood loss [88].

Any type of pelvic inflammation is known as pelvic inflammatory disease (PID), and it is usually the result of an infection in any of the reproductive organs, such as the cervix, ovaries, fallopian tubes, uterine lining, and/or vaginal cavity. The inflammation is caused mostly by bacterial or microscopic parasite infection, which is the most prevalent cause of abnormal vaginal discharge in women; *Chlamydia trachomatis* and *Neisseria gonorrhoeae* are the two most frequent organisms linked with PID [89]. Symptoms of infection include: itching, soreness, and redness around the reproductive organ and grey or yellow vaginal discharge. Long-term complications of PID includes ectopic pregnancy, infertility, and chronic pelvic pain [89]. In a study by Mueller et al., they reported that δ–TCT, which has cholesterol-lowering properties, has the potential to treat *Chlamydia* infection in human because *Chlamydia* species enter the cells through cholesterol-rich lipid raft domains involved in cholesterol trafficking. This claim was supported with their findings, when *Chlamydia* in human lymphocytes was inhibited by 2.6-fold in 1.5 days δ–TCT pre-treated cells [90]. Additionally, this study reveals that TCT may reduce *Chlamydia* growth by lowering the cholesterol, which is essential for its infectivity and replication.

All in all, supplementation with vitamin E has been demonstrated to be effective in the treatment of diseases in women with gynaecological problems. However, more in-depth research is necessary to have a better knowledge of how it works at the molecular level.

## 6. Roles of Vitamin E in Gynaecologic Cancer

The types of gynaecological cancer are cervical, ovarian, uterine, vaginal, and vulvar; the two most common are cervical and ovarian cancer. Ovarian cancer is also the fifth most prevalent cause of cancer mortality among women in the United States (US). According to the American Cancer Society (ACS, Atlanta, GA, USA), they estimated that 214,100 women in the U.S. will be diagnosed with ovarian cancer, with approximately 13,770 American dying from it; around 14,480 women in the U.S. will be diagnosed with cervical cancer, whereas roughly around 4290 American will die of the disease this year. Hence, alternative treatment for these cancers is greatly needed.

Vitamin E has been long-known as a potent agent in preventing the progression of numerous types of cancer, including breast [91,92], lung [93,94], and prostate cancer [95,96]. Numerous in vivo and in vitro studies have shown that vitamin E can directly target cancer cells by inducing apoptosis and decreasing oncogene expression by regulating several signalling pathways, including cyclo-oxygenase (COX)– and 5-lipoxygenase (5-LOX)–catalysed eicosanoids, and transcription factors, such as nuclear transcription factor κB (NF-κB), signal transducer, and activator of transcription factor 3 (STAT3) [97]. In addition to that, some studies suggested that vitamin E is able to modulate immune responses, hence contributing to cancer prevention [47,97,98,99,100]. While vitamin E has been studied extensively for cancer prevention and progression, there are relatively few studies on the effectiveness of vitamin E on gynaecologic cancers (Table 4).

## 7. Conclusions and Future Recommendation

**Figure 1:**
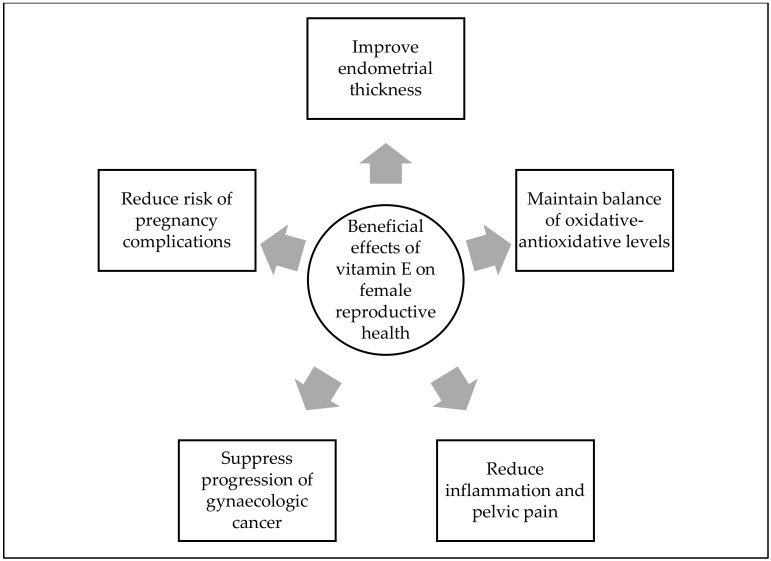
Summary of roles of vitamin E in gynecological health and reproductive-related diseases.

In conclusion, vitamin E supplementation has been demonstrated to have beneficial effects on gynaecological health and reproductive-related diseases (Figure 1). Even though the mechanisms of action of vitamin E have been discovered in several gynaecological diseases, the present literature provides insufficient data on the underlying molecular mechanism of it. Therefore, more comprehensive research is needed before vitamin E can be used for women’s gynaecological health and for managing or preventing gynaecological diseases. Moreover, the majority of the studies on the effects of vitamin E have been conducted using cell lines and animal models; thus, its effects in humans must be thoroughly established before vitamin E can be administrated. Apart from that, several studies failed to provide information on the source, isoform, and ratio of vitamin E utilised in their study, which is crucial for other researchers to refer to when designing or conducting future investigations. Overall, more human evidence-based clinical trials using vitamin E are necessary to back up earlier findings on the benefit of vitamin E supplementation on female fertility and its potential to reduce inflammation and oxidative stress in the female reproductive system.

## Figures and Tables

**Table 1:** Structural isomers of vitamin E.

Type of Vitamin E		Chemical Structure	Type of Sidechain	Number of Methyl Group on Chromanol Ring	Position of Methyl Group on Chromanol Ring
Tocopherols	α	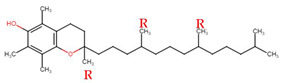	Saturated 16-carbon isoprenoid sidechain	three	5,7,8-trimethyl
	α-tocopheryl acetate	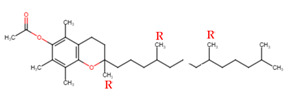			
	β	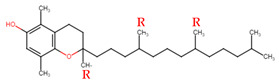		two	5,8-dimethyl
	γ	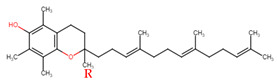		two	7,8-dimethyl
	δ	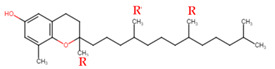		one	8-methyl
Tocotrienols	α	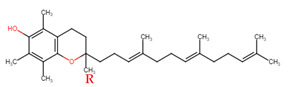	Unsaturated 16-carbon isoprenoid sidechain, containing three double bonds	three	5,7,8-trimethyl
	β	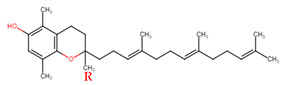		two	5,8-dimethyl
	γ	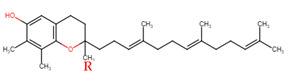		two	7,8-dimethyl
	δ	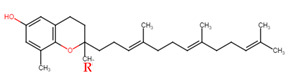		one	8-methyl

**Table 2:** Recommended dietary allowances of vitamin E (α-tocopherol).

Age	RDA in mg (IU)
0–6 months	4 (6)
7–12 months	5 (7.5)
1–3 years	6 (9)
4–8 years	7 (10.4)
9–13 years	11 (16.4)
>14 years	15 (22.4)
Pregnant women	15 (22.4)
Breastfeeding women	19 (28.4)

RDA = recommended dietary allowances; IU = international units. Source: Rivzi et al., 2014 [4].

**Table 3:** Tocopherols and tocotrienols content in different vegetable oils.

	Tocopherols, µg/mL			Tocotrienols, µg/mL		
Sample	α	γ	δ	α	γ	δ
Palm oil	198	-	11	210	408	87
	232	-	9	237	425	78
Sunflower oil	765	-	-	-	-	-
	710	-	-	-	-	-
Cocoa butter	14	225	37	9	-	-
	9	187	31	7	-	-
Walnut oil	12	517	61	-	-	-
	15	569	72	-	-	-
Coconut oil	3	-	13	8	32	-
	5	-	15	11	27	-
Hazelnut oil	425	68	17	-	-	-
	478	74	14	-	-	-
Corn oil	263	1365	88	-	-	-
	245	1319	63	-	-	-

Source: Bonvehi et al., 2000 [35].

**Table 4:** Summary of finding on effect of vitamin E and in female gynaecology cancer.

Type of Vitamin E	Source	Dose	Duration	Type of Cancer	Type of Cell/Tissue	Mechanism	References
α-TCP γ-TCT	Palm oil	150 μM of γ-TCT and 300 μM α-TCP	24 h	Cervix	CaSki	α-TCP and γ-TCT triggered apoptosis via upregulation of p53, Bcl-2-associated X (Bax), and Caspase-3 proteins, as well as Caspase-3 activity.	[101]
α-TCP α-TCT γ-TCT δ-TCT	α-TCP from vegetable oil and TCTs from palm oil	3 μM of each isomer	24 h	Cervix	HeLa	α-TCT and γ-TCT induced apoptosis via cell cycle arrest at G2/M phase in a dose- and time-dependent manner and exerted anti-proliferative properties by increasing the expression of IL-6 and decreasing the expression of cyclin D3, p16, and CDK6 expression in the cell cycle signaling pathway.	[102]
γ-TCT *α*-TCP	Palm oil	150 μM of γ-TCT and 300 μM α-TCP	0, 1, 3, 6, 12, 18, and 24 h	Cervix	CaSki	γ-TCT exerted anti-proliferative properties by suppressing the expression of MEK-2 and ERK-2 proteins.	[103]
γ-TCT	Palm oil	0.5, 1.0, 2.5, and 5.0 μg/mL	7–8 days, until spheres formed	Cervix	HeLa	γ-TCT prevents the development of spherical cervical cancer cells.	[104]
γ-TCT	Palm oil	15, 30, 45, and 60 µM	12, 24, and 48 h	Cervix	HeLa	γ-TCT reduced proliferative cell nuclear antigen (PCNA) and Ki-67 expression and induced apoptosis by reducing the Bcl-2 levels, increasing Bax levels, and release of cytochrome from mitochondria, as well as activating the caspase-9 and caspase-3 activities and ensuing cleavage of poly (ADP-ribose) polymerase (PARP).	[105]
d-*α* tocopheryl acetate (ester of Acetic acid and α-TCP)	Synthetic	0–100 IU	0–72 h	Ovary	Normal and malignant ovarian Surface epithelial (OSE)	d-*α* tocopheryl acetate inhibited cancer cell proliferation via upregulation of caspase-3 activity. Downregulation of hTERT-mRNA Transcription and hTERT promoter activity, thus, blocked the activity of endogenous telomerase.	[106]
α-TCT γ-TCT δ-TCT Cyclophosp-hamide (CPA)	Palm oil	60 mg/kg of TCTs and 10 mg/kg of CPA	Treatment was given for 30 consecutive days.	Ovary	Mice ovarian tissues	Concurrent administration of both TRF and CPA confer protection from apoptosis in ovaries with chemotherapy-induced damage. TCTs administration restored CPA’s harmful effects, which included aberrant folliculogenesis, with decreased ovulation rate, follicular edoema, increased vascularity, and inflammatory cell infiltration.	[107,108]
δ-TCT bevacizumab	Annatto	300 mg orally, three times daily	The treatment continued until progression, grade 3 toxicity, or patient wish to discontinue.	Ovary	Human clinical trial	Concurrent administration of δ-TCT with bevacizumab indicate an additive effect in chemotherapy refractory cancer.	[109]

## Data Availability

Not applicable.
